# TiO_2_, GO, and TiO_2_/GO Coatings by APPJ on Waste ABS/PMMA Composite Filaments Filled with Carbon Black, Graphene, and Graphene Foam: Morphology, Wettability, Thermal Stability, and 3D Printability

**DOI:** 10.3390/polym17243263

**Published:** 2025-12-09

**Authors:** Alejandra Xochitl Maldonado Pérez, Alma Delfina Arenas Flores, José de Jesús Pérez Bueno, Maria Luisa Mendoza López, Yolanda Casados Mexicano, José Luis Reyes Araiza, Alejandro Manzano-Ramírez, Salomón Ramiro Vásquez García, Nelly Flores-Ramírez, Carlos Montoya Suárez, Edain Belén Pérez Mendoza

**Affiliations:** 1 Centro de Investigación y Desarrollo Tecnológico en Electroquímica, S.C., Parque Tecnológico Sanfandila, Pedro Escobedo 76703, Querétaro, Mexico; ad.arenasf@gmail.com (A.D.A.F.); ycasados03@gmail.com (Y.C.M.); cmontoya@cideteq.mx (C.M.S.); 2 Tecnológico Nacional de México, Instituto Tecnológico de Orizaba, Oriente 9, Emiliano Zapata, Orizaba 94320, Veracruz, Mexico; 3 Tecnológico Nacional de México, Instituto Tecnológico de Querétaro, Av. Tecnológico s/n Esq. M. Escobedo, Col. Centro, Santiago de Querétaro 76000, Querétaro, Mexico; maria.ml@queretaro.tecnm.mx; 4 Facultad de Ingeniería, Universidad Autónoma de Querétaro, Cerro de las Campanas s/n Cto. Universitario, Centro Universitario, Santiago de Querétaro 76010, Querétaro, Mexico; araiza@uaq.edu.mx; 5 Centro de Investigación y de Estudios Avanzados del IPN, Unidad Querétaro, Libramiento Norponiente #2000, Fracc. Real de Juriquilla, Santiago de Querétaro 76230, Querétaro, Mexico; amanzano@cinvestav.mx; 6 Facultad de Ingeniería Química, Universidad Michoacana de San Nicolás de Hidalgo, Morelia 58030, Michoacán, Mexico; salomon.vasquez@umich.mx; 7 Facultad de Ingeniería en Tecnología de la Madera, Universidad Michoacana de San Nicolás de Hidalgo, Morelia 58030, Michoacán, Mexico; nelly.flores@umich.mx; 8 Tecnológico de Monterrey, Epigmenio González 500, Fracc. San Pablo, Santiago de Querétaro 76130, Querétaro, Mexico; a01706834@tec.mx

**Keywords:** waste thermoplastics, ABS, PMMA, graphene-based fillers, TiO_2_/GO coatings, atmospheric pressure plasma jet

## Abstract

This work presents a multifactorial strategy for reusing waste thermoplastics and generating multifunctional filaments for additive manufacturing. Acrylonitrile–butadiene–styrene (ABS) waste and commercial poly(methyl methacrylate) (PMMA) were compounded with carbon black (CB), graphene (G), or graphene foam (GF) at different loadings and extruded into composite filaments. The aim is to couple filler-induced bulk modifications with atmospheric pressure plasma jet (APPJ) surface coatings of TiO_2_ and graphene oxide (GO) to obtain waste-derived filaments with tunable morphology, wettability, and thermal stability for advanced 3D-printed architectures. The filaments were subsequently coated with TiO_2_ and/or GO using an APPJ process, which tailored surface wettability and enabled the formation of photocatalytically relevant interfaces. Digital optical microscopy and SEM revealed that CB, G, and GF were reasonably well dispersed in both polymer matrices but induced distinct surface and cross-sectional morphologies, including a carbon-rich outer crust in ABS and filler-dependent porosity in PMMA. For ABS composites, static contact-angle measurements show that APPJ coatings broaden the apparent wettability window from ~60–80° for uncoated filaments to ~40–50° (TiO_2_/GO) up to >90° (GO), corresponding to a ≈150% increase in contact-angle span. For PMMA/CB composites, TiO_2_/GO coatings expand the accessible contact-angle range to ~15–125° while maintaining surface energies around 50 mN m^−1^. TGA/DSC analyses confirm that the composites and coatings remain thermally stable within typical extrusion and APPJ processing ranges, with graphene showing only ≈3% mass loss over the explored temperature range, compared with ≈65% for CB and ≈10% for GF. Fused deposition modeling trials verify the printability and dimensional fidelity of ABS-based composite filaments, whereas PMMA composites were too brittle for reliable FDM printing. Overall, combining waste polymer reuse, tailored carbonaceous fillers, and APPJ TiO_2_/GO coatings provides a versatile route to design surface-engineered filaments for applications such as photocatalysis, microfluidics, and soft robotics within a circular polymer manufacturing framework.

## 1. Introduction

Polymer processing is one of the most versatile and scalable routes for developing advanced materials, since extrusion enables the melt blending of thermoplastic matrices with fillers and additives to tailor structural and functional properties at laboratory, pilot, and industrial scales [1,2]. Acrylonitrile–butadiene–styrene (ABS) is a widely used engineering thermoplastic due to its rigidity, impact resistance, chemical stability, and excellent surface finish, properties that also make it attractive for fused filament fabrication (FFF) and other extrusion-based additive manufacturing processes [3]. Conventionally, ABS has been used as an electrical insulator, but the incorporation of conductive carbonaceous fillers, such as carbon black (CB), can expand its use toward antistatic and electromagnetic shielding components [4].

In addition to imparting electrical conductivity, fillers such as CB, graphene (G), and graphene foam (GF) can serve as pigments and mechanical reinforcements. In poly(methyl methacrylate) (PMMA) and related systems, carbonaceous nanostructures have been shown to enhance UV resistance and modify mechanical response by reducing photooxidation and acting as load-bearing phases [5,6,7]. Graphene-based materials, in particular, offer exceptional electronic, mechanical, and chemical properties owing to their two-dimensional honeycomb lattice [8,9]. Numerous studies have demonstrated that graphene and its derivatives affect pyrolysis behavior, thermal conductivity, melting and dripping characteristics, and viscosity of polymer composites, enabling applications in the automotive sector, construction materials, household appliances, and electrical devices [10].

For 3D printing, graphene-filled thermoplastics have been designed to simultaneously improve thermal conductivity, melt behavior, and macroscopic performance of printed parts. Chiu et al. patented a graphene-reinforced polymer composite with uniformly dispersed graphite and multi-layer graphene nanoparticles, reporting improved structural and thermal properties at loadings of 10–50 wt% [11]. Singh et al. showed that ABS–graphene filaments processed by FDM can reach enhanced electrical and thermal conductivity without sacrificing mechanical performance [12]. Similar trends have been reported for carbon nanotubes (CNT)- and graphene-filled filaments, where filler content and rheology must be tuned to reconcile printability with functionality [4,13].

In parallel, TiO_2_-assisted surface modification of ABS has been used to enable metallization and photocatalytic functionality without aggressive chromic-acid etching [14,15,16]. These works demonstrate that oxide coatings can be integrated directly onto thermoplastic components, opening opportunities for hybrid structural–functional parts. Additive manufacturing further expands this potential by enabling the fabrication of complex, customized geometries from functionalized or pigmented filaments with specific mechanical, electrical, or surface properties [17,18,19,20,21]. For instance, TiO_2_–ABS nanocomposite filaments have been printed into photocatalytically active components [22], and fully recycled TiO_2_–polystyrene filaments have been used to produce 3D-printed photocatalysts for degrading pharmaceutical residues [23]. Moderate graphene oxide (GO) loadings in ABS filaments have also been shown to improve stiffness and FDM print quality [24]. In parallel, recent work on silane-modified Al_2_O_3_ nanoparticle–reinforced epoxy composites has demonstrated that tailoring filler surface chemistry and dispersion can increase tensile strength and hardness by several tens of percent relative to the neat matrix [25]. Beyond filament-based systems, TiO_2_ photocatalytic reactors have been fabricated by stereolithography (SLA) and digital light processing (DLP), where the interplay between reactor geometry and TiO_2_ distribution governs photocatalytic performance [26,27].

More broadly, extrusion-based additive manufacturing, including FFF and bioprinting, has illustrated how coupling tailored filler systems with appropriate rheology and processing windows can yield multifunctional 3D constructs [13,28,29,30]. However, three aspects remain only partially explored in the context of waste-derived thermoplastics. First, the reuse of waste ABS and PMMA as matrices for graphene- and CB-based composites has rarely been combined with a systematic analysis of morphology, wetting, and thermal behavior. Second, most APPJ-deposited TiO_2_/GO coatings have been studied on flat substrates [30], rather than directly on filament geometries where curvature and roughness complicate conformal coverage. Third, integrated studies that correlate bulk morphology and thermal stability with surface wettability and 3D printability within a single, waste-based filament platform are scarce. Addressing these gaps is relevant for circular-economy strategies in additive manufacturing, where recycled feedstocks are upgraded into surface-engineered, application-oriented filaments rather than simple structural materials [3,23,31,32,33].

In this work, a multifactorial approach is implemented to develop multifunctional polymer filaments by reusing waste thermoplastics and tailoring their properties through hybrid filler reinforcement and advanced surface modification. Waste ABS and commercial PMMA are compounded with CB, G, or GF at different loadings by twin-screw extrusion to tune bulk electrical, mechanical, and thermal characteristics. The extruded filaments are then coated with TiO_2_ and/or GO using an atmospheric pressure plasma jet (APPJ) process, which simultaneously modifies surface wettability and enables photocatalytically active interfaces [30]. This strategy decouples bulk mechanical performance from surface functionality by placing TiO_2_-based and GO-based coatings on the outer surface of waste-derived cores, rather than dispersing oxides throughout the bulk [22,23]. The morphological distribution of carbonaceous fillers is assessed by digital optical microscopy and SEM, while calorimetric and wettability measurements confirm homogeneous dispersion within the chosen processing window and tunable surface energy across formulations. The printability and dimensional stability of the coated filaments are validated via FDM of representative prototypes. Overall, the approach integrates five key aspects: (i) reuse of waste polymers, (ii) optimization of filament bulk properties via carbonaceous fillers, (iii) precise additive manufacturing of complex 3D designs, (iv) functional surface modification with TiO_2_ and/or GO thin films, and (v) automated nanoparticle deposition via APPJ to produce consistent, multifunctional coatings. Compared with previous reports on ABS–graphene, TiO_2_–ABS, and recycled TiO_2_–polymer filaments, which typically focus either on bulk mechanical/electrical performance or on photocatalytic activity alone, the present work uniquely combines waste-derived ABS/PMMA cores, three types of carbonaceous fillers, and APPJ TiO_2_/GO surface coatings while systematically correlating morphology, wettability, thermal stability, and FDM printability within a single filament platform.

## 2. Materials and Methods

### 2.1. Materials and Polymeric Mixture Preparations

ABS and PMMA were employed as polymer matrices for filament production. Carbon black, graphene, and graphene foam were used as carbonaceous fillers. For surface multifunctional modification, TiO_2_ and GO were utilized as coating materials. All chemicals were used as received without further purification.

ABS and PMMA were employed as polymer matrices for filament production. Waste ABS originated from injection-molding purges (Reciclajes Victoria, Santiago de Querétaro, México), and commercial PMMA pellets (MIDSA, Nuevo León, México) were used as received. Carbon black (CB, ID-Nano, San Luis Potosí, México), graphene (G, ID-Nano, San Luis Potosí, México), and graphene foam (GF, ID-Nano, San Luis Potosí, México) served as carbonaceous fillers. According to the manufacturer, the graphene nanoplatelets consist of platelet-like flakes and sub-micrometric thickness, whereas the graphene foam particles exhibit a 3D carbon structure. For surface multifunctional modification, TiO_2_ nanoparticles (Degussa P-25—Evonik Aeroxide^®^ TiO_2_ P 25; Hanau, Germany) and graphene oxide (GO, ID-Nano, San Luis Potosí, México) were utilized as coating materials. All chemicals were used as received without further purification.

ABS waste material was first ground using a Retsch PM-100 ball mill. Grinding was performed using a 5.5 kg stainless steel vial containing four 10 mm and four 20 mm stainless steel balls. About 230 g of ABS was processed for 10 min at 300 rpm. Subsequently, the ground ABS was mixed with carbon black, graphene, or graphene foam at filler loadings of 0.5, 1, and 2 wt%. Before extrusion, the ground ABS and PMMA mixtures were dried in a convection oven at 80 °C for 2 h to ensure effective moisture removal.

**Figure 1:**
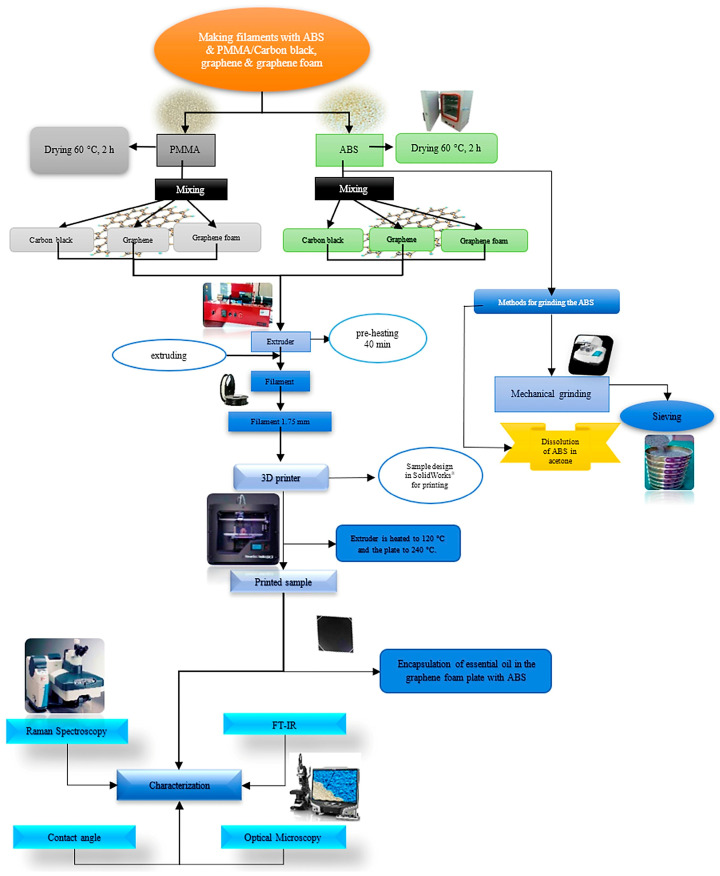
Workflow for preparing waste polymer composite filaments. Grinding of ABS waste and compounding with carbon black (CB), graphene (G), or graphene foam (GF). Manual mixing of PMMA with the same fillers, followed by drying of both material streams before filament extrusion.

PMMA was manually mixed with CB, graphene, or GF at the same filler concentrations (0.5, 1, and 2 wt%). No additional grinding step was required for PMMA because the as-received pellets already had a suitable size distribution for compounding. The overall sustainable workflow for recovering waste ABS, compounding both polymers with the different carbonaceous fillers, and preparing them for extrusion is summarized in Figure 1. The scheme highlights the parallel processing routes for ABS and PMMA, the points at which filler type and loading are selected, and the pre-drying step before extrusion, all of which are essential for interpreting the subsequent morphological, wettability, and thermal results.

**Figure 2:**
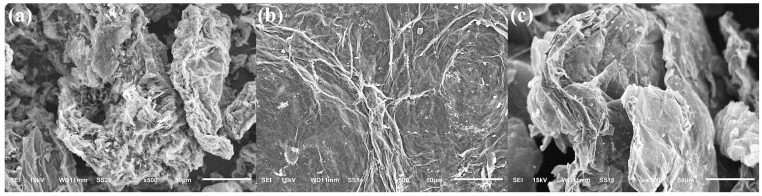
SEM micrographs of the as-received carbonaceous fillers used in this work: (**a**) graphene (G), (**b**) graphene oxide (GO), and (**c**) graphene foam (GF).

The as-received morphologies of the three carbonaceous fillers are shown in Figure 2a–c of graphene, graphene oxide, and graphene foam powders, respectively. All images were acquired at 15 kV and 500× magnification, with a scale bar of 50 µm. Graphene and GO appear as wrinkled, platelet-like aggregates with lateral dimensions of a few tens of micrometers, whereas graphene foam shows a more open, three-dimensional network with interconnected walls and pores.

### 2.2. D Printing of Test Specimens and Demonstrator Components

**Figure 3:**
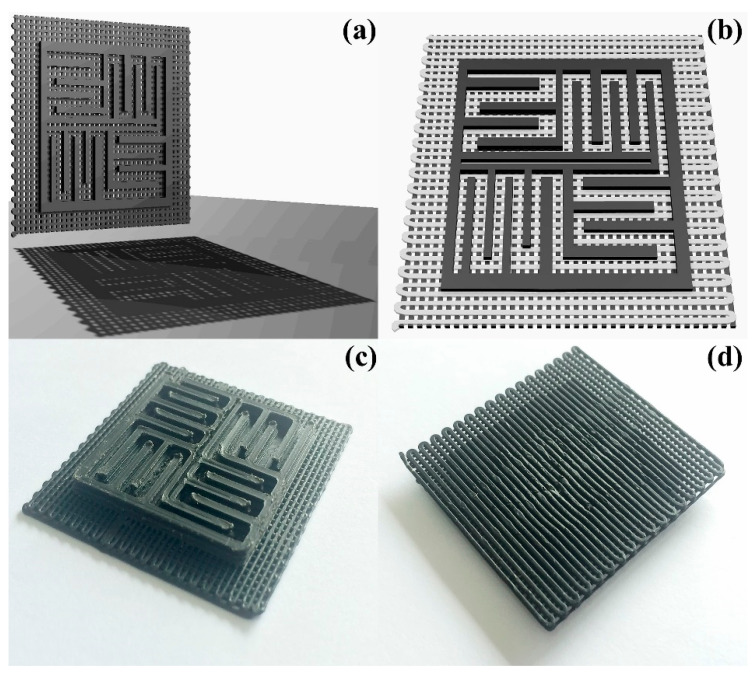
Prototype used to evaluate the printability of the composite filaments. (**a**,**b**) CAD model of the 3 × 3 cm stepped geometry created in SolidWorks^®^. (**c**,**d**) top and bottom views of the 3D-printed component fabricated with the ABS/CB filament.

CAD models used for the printing trials were created in SolidWorks^®^ (Premium 2022 SP1.0). The primary test geometry consisted of a 3 × 3 cm square-based specimen with a stepped profile, designed to highlight layer stacking, edge definition, and dimensional fidelity. Additionally, more complex prototypes were generated to illustrate the capability of the composite filaments to produce non-trivial three-dimensional architectures (Figure 3).

The composite filaments were fed into a commercial FDM 3D printer [34]. Before each print, the nozzle and build plate were preheated for 15 min to ensure stable melt flow and adequate adhesion. The extruder was primed by manually feeding filament through the nozzle to prevent clogging. Printing was carried out with a nozzle diameter of 1 mm, a layer height of 1 mm, and a linear print speed of approximately 0.4 mm/s, as recommended by the manufacturer for ABS-based materials. Under these conditions, the pieces were built as stacked layers following the toolpath defined by the CAD model, from the base upward until the complete geometry was formed.

Filaments were extruded using a Noztek Pro single-screw extruder with a die diameter of 2.0 mm, which is compatible with the 3D printer (MakerBot Replicator 2X). The barrel and die temperatures were set to 150–160 °C (±10 °C) for PMMA and 240–250 °C (±10 °C) for ABS. The extrusion rate was ≈2–3 cm/min (≈0.3–0.5 mm/s), resulting in filament lengths of 50–70 cm over 20–30 min of continuous operation. The extruded filaments were subsequently used in a fused deposition modeling (FDM) printer, where the nozzle and build plate temperatures were set within the standard operating window for ABS-based filaments to ensure stable melt flow, adequate adhesion between successive layers, and dimensional stability. Typical values for these prints were in the range of 230–250 °C at the nozzle and 100–110 °C at the build plate, which are high enough to promote polymer diffusion across layer interfaces but remain below the onset temperatures of thermal degradation identified by TGA/DSC. These printing settings were maintained constant for all ABS-based composites, allowing the influence of filler type and concentration on printability to be evaluated under comparable FDM conditions.

Preliminary FDM trials performed under the same conditions used for ABS-based filaments showed that PMMA-based composite filaments fractured repeatedly in the feeding path and at the drive gear–nozzle transition, typically after only a few centimeters of extrusion. This prevented the continuous deposition of full test pieces. Because no dedicated mechanical optimization of the PMMA formulations was attempted here, systematic 3D printing experiments were therefore restricted to ABS-based composites with CB, G, or GF fillers. The resulting prototypes (Figure 3) were used to qualitatively assess surface finish, dimensional stability, and layer resolution. The good agreement between the CAD geometry (Figure 3a,b) and the printed component (Figure 3c,d) demonstrates that filaments produced from waste ABS and carbonaceous fillers can be effectively processed by FDM and serve as demonstrator parts for potential applications such as microfluidic manifolds, soft robotic elements, or flexible electronic supports, in line with recent reports on graphene-oxide-based 3D-printed devices [35].

### 2.3. Surface Coating with TiO_2_ and GO

Surface covering of the extruded filaments was carried out using an APPJ system. TiO_2_ and GO coatings were applied sequentially. For the GO coating, a suspension was prepared by dispersing 0.02 g/L of GO in deionized water. The suspension was sonicated for 3 h in an ultrasonic bath, then stirred for 30 min at 250 rpm to ensure homogeneity. For TiO_2_ (P25, Degussa), a suspension of 0.02 g/L was similarly prepared, sonicated for 45 min, and stirred for 30 min at 250 rpm. The selected APPJ operating parameter values (voltage, gas flow, and nozzle–substrate distance) fall within the previously reported range for conformal TiO_2_ coatings on polymer substrates, without thermally damaging the thermoplastic core [36].

The coatings were applied using a nebulizer to generate a mist of the solution, with the nozzle directed toward the plasma plume. A pretreatment step using the plasma jet alone was performed to clean the filament surface and make it more hydrophilic, thereby reducing the contact angle and enhancing coating uniformity. The plasma jet was operated continuously, interspersed with the application of a solution mist for coating deposition. This process was conducted for 2 min per layer, which was deemed an adequate time. TiO_2_ coatings were applied first, followed by the GO layer. Other deposition parameters were kept unchanged, such as the standoff distance of approximately 3 cm from the nozzle or 0.5 cm from the plasma plume. The output plasma power was approximately 2 kVA, and the air gas flow rate was 2000 L h^−1^ with an inlet pressure of 4.9 bar. The environmental conditions were a room temperature of about 20 °C and a relative humidity of about 40%. The present APPJ configuration and precursor delivery system follow those previously optimized for self-assembled GO coatings, with minor adjustments in nozzle–substrate distance and scan speed [30]. In this work, the nozzle was rastered along the filament at a scan speed of about 140 mm s^−1^ (corresponding to a linear translation of 840 cm min^−1^), ensuring overlap between consecutive passes without overheating or visibly deforming the thermoplastic core. The effective deposition time on any surface site was about 2 min.

### 2.4. Characterization Techniques

The surface morphology and filler dispersion in the filaments were examined using a Keyence VHX-5000X digital microscope (Keyence, Osaka, Japan). Cross-sectional views were prepared to assess the internal distribution of filler.

Static contact angles and surface free energies were measured at room temperature using a Krüss DSA30 drop shape analyzer. Sessile drops of 3–5 µL of deionized water and diiodomethane were deposited on the filament surface using an automatic dosing unit; for each formulation and coating, 3–5 droplets were placed at different positions along the filament, and both left and right contact angles were obtained by fitting the droplet profile. In this configuration, the static contact angle θ is defined as the angle between the liquid–vapor interface and the solid surface at the three-phase contact line, reflecting the balance between adhesive liquid–solid interactions and cohesive forces within the liquid. Low θ values (<90°) indicate hydrophilic, high-surface-energy interfaces, whereas high θ values (>90°) correspond to more hydrophobic, low-surface-energy surfaces. The surface free energy was estimated using the Owens–Wendt–Rabel–Kaelble method, based on the contact angles of water and diiodomethane. Measurements were performed on both coated and uncoated filaments to quantify how filler type and APPJ-deposited TiO_2_/GO layers modify the wetting response.

Thermal stability and phase transitions were characterized by thermogravimetric analysis (TGA) and differential scanning calorimetry (DSC) using a NETZSCH STA 449F5 instrument (Selb, Germany). Samples (~4 mg) were placed in aluminum crucibles and heated at a ramp rate of 10 °C/min under an oxygen flow of 50 mL/min.

## 3. Results and Discussion

Before analyzing the polymer–filler composites, the morphology of the carbonaceous fillers was characterized by SEM (Figure 2). Graphene (Figure 2a) consists of wrinkled, multi-layer aggregates in which stacks of plate-like flakes form porous clusters with lateral dimensions on the order of several tens of micrometers. Graphene oxide (Figure 2b) displays larger, sheet-like regions with smoother areas and occasional folds or cracks, consistent with partially exfoliated oxide platelets. Fragments of the graphene foam (Figure 2c) retain an open, cellular configuration with curved walls and pores in the 10–50 µm range, reflecting the three-dimensional network nature of the foam. These distinct morphologies anticipate different interactions with the ABS and PMMA matrices: graphene and GO are expected to behave as high-aspect-ratio platelets that can align or restack under shear, whereas GF provides a more rigid, skeletal framework that can generate localized porosity and roughness in the composites.

### 3.1. PMMA Filaments

**Figure 4:**
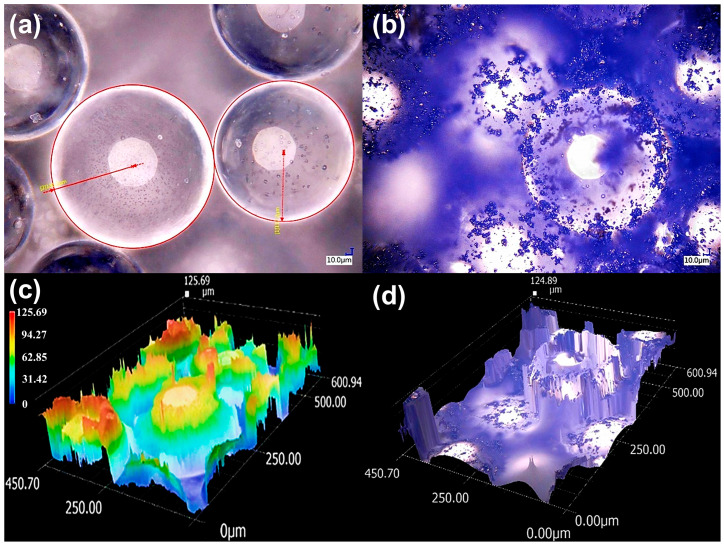
Digital optical microscopy of PMMA and PMMA/CB composites. (**a**) Pristine PMMA spheres and (**b**) PMMA containing 1 wt% carbon black (CB), where CB particles decorate the polymer surface without large agglomerates. (**c**,**d**) 3D surface reconstructions of (**a**,**b**), respectively.

Figure 4 compares the morphology of pristine PMMA particles and PMMA particles containing 1 wt% CB before filament extrusion. The PMMA spheres exhibit radii of approximately 135 and 110 µm, resulting in a relatively narrow size distribution (Figure 4a). When CB is added (Figure 4b), the carbonaceous particles preferentially decorate the PMMA surface, producing dark patches rather than large agglomerates. At this stage, the polymer is not uniformly black, but the filler distribution is reasonably homogeneous at the microscale. The 3D surface reconstructions (Figure 4c,d) reveal an increase in surface roughness when CB is present, consistent with the formation of local protrusions and depressions associated with the pigment particles.

**Figure 5:**
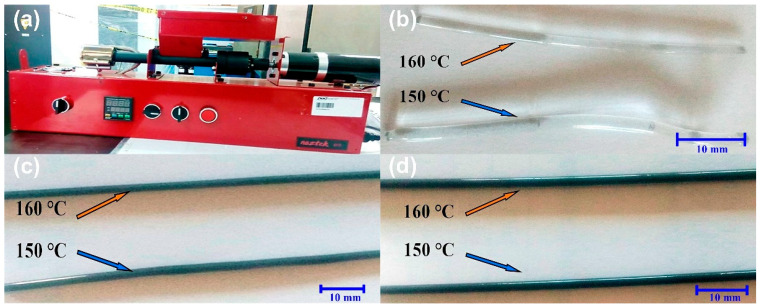
Macroscopic appearance of extruded PMMA filaments with increasing carbon black (CB) content. (**a**) Extrusion of PMMA/CB composites in the Noztek Pro single-screw extruder. (**b**–**d**) Filaments containing 0, 0.5, and 1 wt% CB, respectively. The scale bar corresponds to 10 mm.

After compounding and extrusion, the macroscopic appearance of the PMMA filaments changes significantly (Figure 5). The recycled PMMA filament without CB retains its characteristic transparency (Figure 5b), whereas the filaments containing 0.5 and 1 wt% CB (Figure 5c,d) exhibit a progressive darkening with filler content. The uniform black coloration along the filament length, without visible streaks or longitudinal bands, indicates satisfactory dispersion and transport of CB during extrusion [9]. From a processing perspective, Figure 5 confirms that waste PMMA and CB can be combined into visually homogeneous filaments, a prerequisite for consistent mechanical and electrical performance. Under the constant extrusion and FDM conditions, increasing the CB content from 0 to 1 wt% did not require adjustments in nozzle temperature, print speed, or extrusion pressure. The filaments remained compatible with the nominal 2.0 mm die diameter, and the printed layers exhibited comparable thickness and stacking quality. Thus, within the explored 0–1 wt% range, CB primarily acts as a pigment and roughness modifier rather than as a limiting factor in filament diameter control or printing stability.

**Figure 6:**
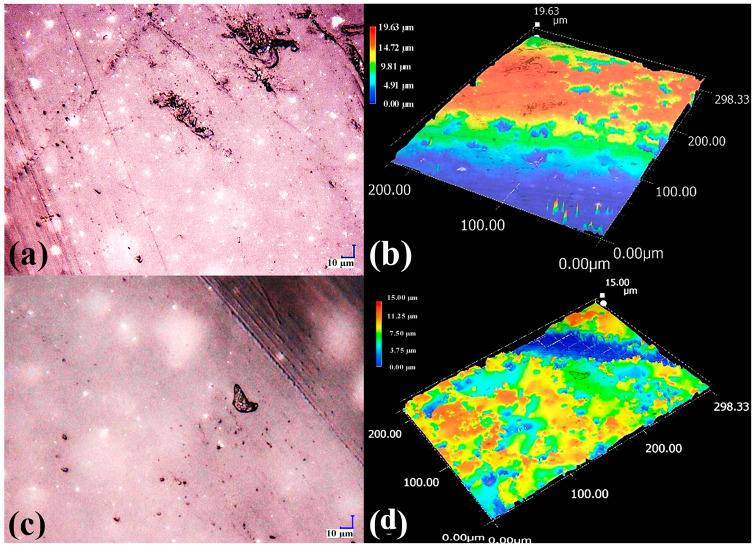
High-magnification (1000×) digital microscopy of PMMA filaments with carbon black (CB). (**a**,**c**) Surface micrographs and (**b**,**d**) corresponding color-scale height maps for filaments with 0.5 and 1 wt% CB, respectively.

The effect of CB loading on filament surface topography is examined in Figure 6. At 1000× magnification, both PMMA/CB compositions show a continuous surface with local defects (Figure 6a,c), but the density and size of microvoids, pits, and rough patches increase for 1 wt% CB. The corresponding color-scale height maps (Figure 6b,d) clearly distinguish elevated features (red–yellow areas) and depressions (blue regions), confirming that CB not only pigments the polymer but also promotes protrusions and valleys at the microscale. Such roughness can be beneficial for subsequent coating adhesion, but may also act as a stress concentrator, reducing mechanical strength if cracks initiate at these defects.

**Figure 7:**
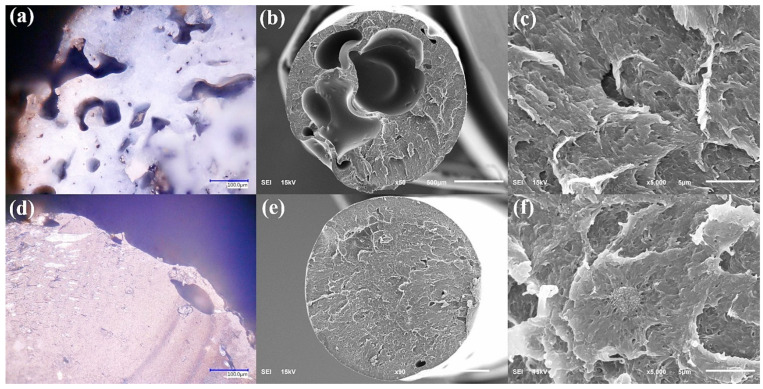
Cross-sectional morphology of PMMA filaments with carbon black (CB) filler. (**a**,**d**) Optical micrographs at 500× of filaments with 0.5 and 1 wt% CB, respectively. (**b**,**c**) SEM images of the 0.5 wt% CB filament at 50× and 5000×, showing a more porous, irregular interior. (**e**,**f**) SEM images of the 1 wt% CB filament at 90× and 5000×, revealing a denser, more homogeneous cross-section.

Cross-sectional analyses of the PMMA filaments (Figure 7) provide complementary information on the internal structure. Optical and SEM images show that filaments with 0.5 wt% CB (Figure 7a–c) exhibit a more porous and irregular cross-section than those containing 1 wt% CB (Figure 7d–f). At lower magnifications (50×–500×), voids and incomplete fusion between strands are more evident in the 0.5 wt% CB filaments, whereas the 1 wt% CB filaments appear denser and more homogeneous. At higher magnification (5000×), both compositions exhibit similar microstructural features, indicating that CB is finely distributed within the PMMA matrix and that the primary differences occur at the mesoscopic scale rather than the nanoscale.

Taken together, Figure 4, Figure 5, Figure 6 and Figure 7 indicate that (i) CB can be dispersed reasonably uniformly on the surface of PMMA particles before extrusion, (ii) extrusion produces macroscopically homogeneous, pigmented filaments (Figure 5), and (iii) increasing CB content tends to reduce large-scale porosity while increasing surface roughness. This combination is relevant to subsequent APPJ coatings and wettability, as surface roughness and internal densification can simultaneously influence coating uniformity, mechanical integrity, and liquid–solid interactions.

### 3.2. ABS Filaments

**Figure 8:**
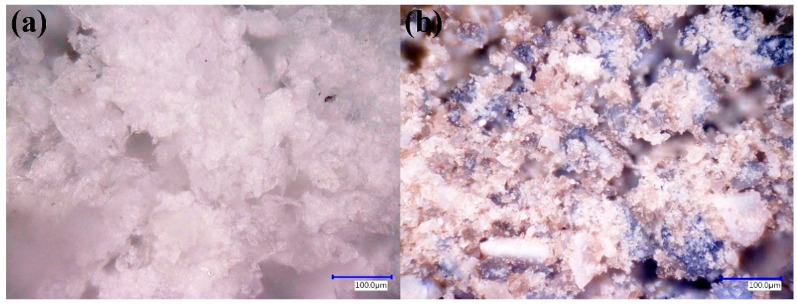
Optical micrographs (500×) of ground ABS composite mixtures before filament extrusion. (**a**) ABS with carbon black (CB) and (**b**) ABS with graphene foam (GF).

The effect of the different carbonaceous fillers on the ABS mixtures before extrusion is shown in Figure 8. The digital micrographs at 500× reveal that the ground ABS mixed with CB (Figure 8a) retains a relatively light background with dark CB particles distributed across the surface, whereas the ABS mixed with GF (Figure 8b) exhibits a darker and more heterogeneous appearance. This suggests that GF interacts more strongly with the polymer fragments, leading to higher apparent pigmentation even before filament formation. The grinding traces of the ABS particles are also visible, confirming that the ball milling step effectively reduces the waste ABS to a size suitable for compounding.

**Figure 9:**
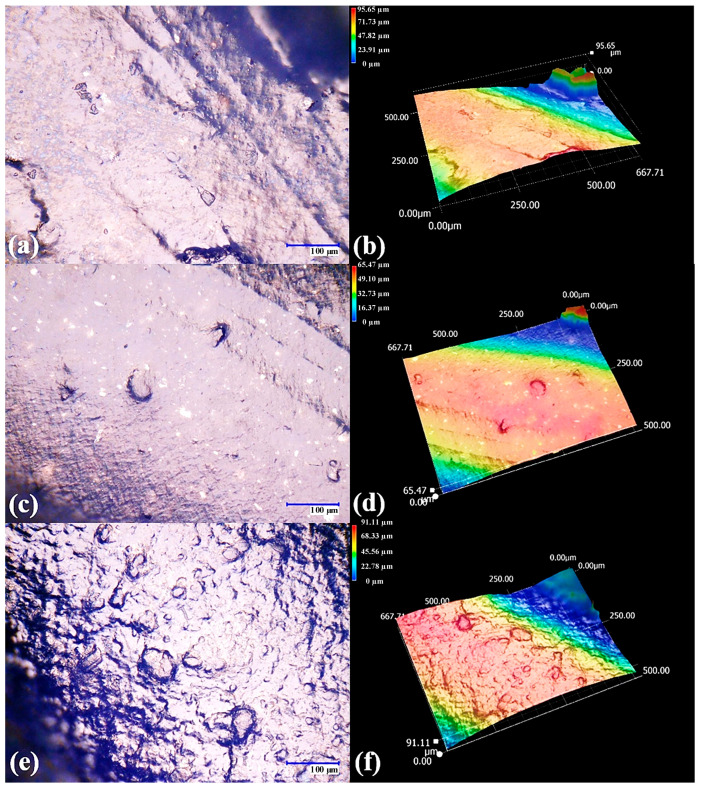
Surface morphology of ABS composite filaments with different carbonaceous fillers. (**a**,**c**,**e**) Optical micrographs and (**b**,**d**,**f**) color-scale height maps for ABS filaments filled with graphene foam (GF), graphene (G), and carbon black (CB), respectively.

Once extruded, the surfaces of the ABS composite filaments display characteristic differences as a function of the filler type (Figure 9). The optical micrographs (Figure 9a,c,e) show that all filaments attain a rounded contour with an approximate diameter of 2 mm, indicating that the extrusion conditions were adequate to form continuous strands. However, the color-scale height maps (Figure 9b,d,f) reveal variations in surface roughness: CB-filled filaments exhibit the largest amplitude of topographical features. In contrast, filaments with graphene and GF are comparatively smoother. The darker surface coloration is consistent with the presence of the carbonaceous fillers and masks the underlying white ABS matrix, in agreement with previous reports on carbon-filled thermoplastics [37].

**Figure 10:**
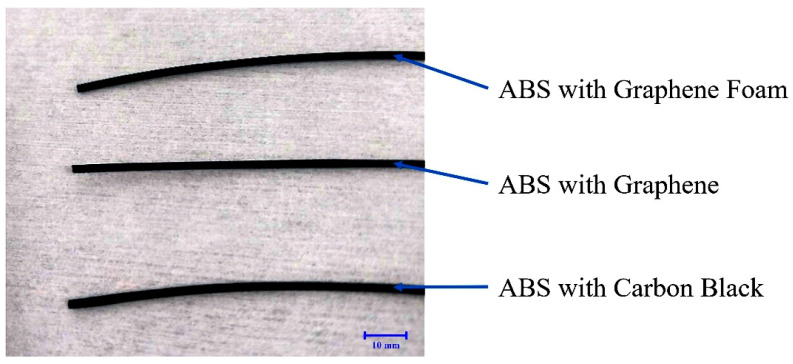
Photographs of extruded ABS composite filaments filled with graphene foam (GF), graphene (G), and carbon black (CB). The scale bar corresponds to 10 mm.

**Figure 11:**
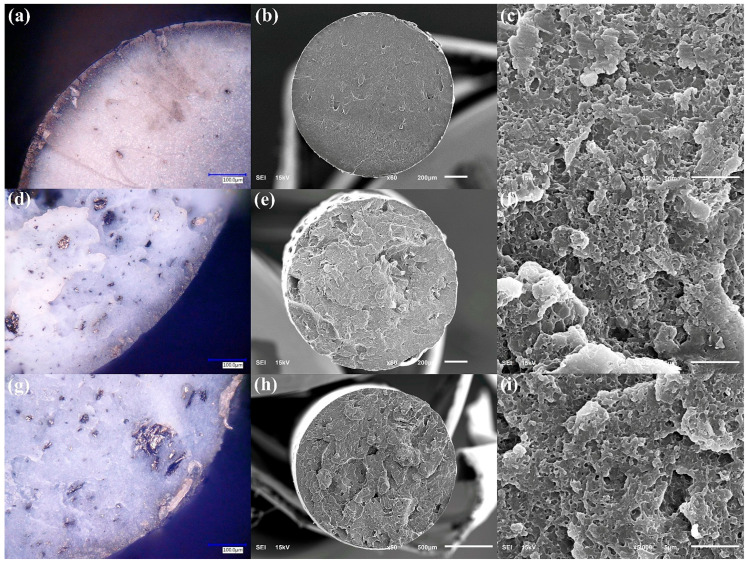
Cross-sectional morphology of ABS composite filaments containing carbonaceous fillers. (**a**,**d**,**g**) Optical micrographs at 500× for ABS/CB, ABS/G, and ABS/GF filaments, respectively, evidencing a dark outer crust encasing a lighter ABS core. (**b**,**c**) SEM images of ABS/GF at 50× and 5000×, (**e**,**f**) ABS/G at 50× and 5000×, and (**h**,**i**) ABS/CB at 50× and 5000×.

Macroscopic photographs of the extruded ABS composite filaments (Figure 10) show that, at the scale of the naked eye, there are no dramatic differences in appearance among filaments containing GF, graphene, or CB. All filaments appear uniformly black with a similar gloss, underscoring the need for microscopic and cross-sectional analyses (Figure 9 and Figure 11) to distinguish between formulations. In practical terms, this also means that the different fillers can be processed into filaments that are visually indistinguishable but possess distinct surface and bulk properties.

Figure 11 provides cross-sectional views of the ABS composite filaments. The optical micrographs at 500× (Figure 11a,d,g) indicate a core–shell-like structure for all compositions: a darker outer crust, enriched in carbonaceous filler, encases a lighter ABS core. This crust is attributed to the combination of the filler distribution and the thermal profile experienced by the filament during extrusion and cooling. The SEM images at 50× and 5000× (Figure 11b,c,e,f,h,i) show the characteristic granular morphology of ABS. At low magnification, ABS/GF filaments present a smoother cross-section than those with graphene or CB, whereas at higher magnification, the three composites display comparable microtextures with embedded filler domains.

Comparing Figure 7 and Figure 10 highlights differences between the PMMA- and ABS-based systems. While PMMA filaments exhibit more elongated, layered features in cross-section (Figure 7), ABS filaments show a grain-like texture and, in all cross-sections analyzed along a given filament, a darker carbon-rich outer band encasing a lighter ABS-rich core (Figure 11). Under the current extrusion conditions, this shell–core structure is reproducible along the filament length, although a complete statistical analysis across multiple extrusion batches is beyond the scope of this work. These distinctions are expected to impact mechanical behavior, thermal conduction, and wettability.

### 3.3. TiO_2_ and GO Coatings

**Figure 12:**
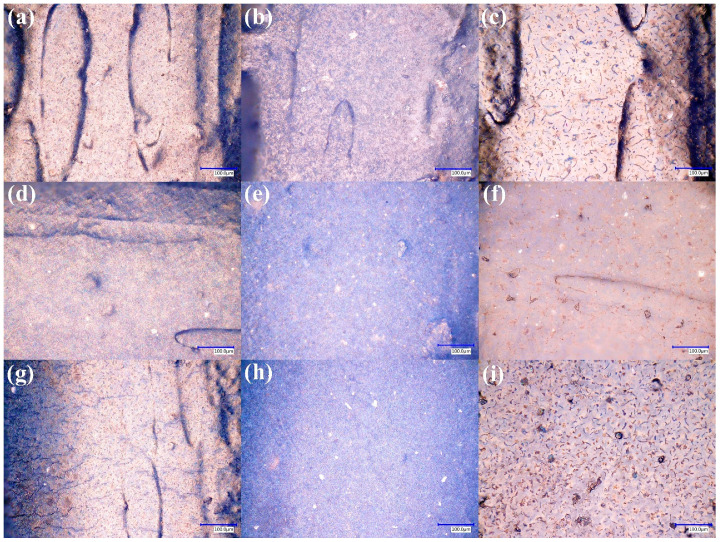
Optical micrographs (500×) of ABS composite filament surfaces after APPJ-based TiO_2_ and GO coatings. Rows correspond to (**a**–**c**) ABS/GF, (**d**–**f**) ABS/G, and (**g**–**i**) ABS/CB filaments. Columns show filaments coated with (**a**,**d**,**g**) TiO_2_/GO, (**b**,**e**,**h**) TiO_2_, and (**c**,**f**,**i**) GO, respectively.

The effect of the APPJ-based surface functionalization on ABS composite filaments is shown in Figure 12. Each row corresponds to a single filler type (GF, graphene, or CB), and each column represents a distinct coating configuration (TiO_2_/GO, TiO_2_, or GO). In all cases, the coatings form continuous layers that conform to the underlying rough surfaces observed in Figure 9, without noticeable pinholes or areas where the bare polymer is exposed. The TiO_2_-containing coatings (Figure 12b,e,h) display the expected whitish appearance associated with the high refractive index of TiO_2_, whereas the GO-only coatings (Figure 12c,f,i) exhibit a more iridescent tone. The combined TiO_2_/GO layers (Figure 12a,d,g) show intermediate contrast, consistent with partial coverage of the TiO_2_ underlayer by GO. These qualitative observations indicate that the APPJ process successfully deposits thin films that continuously follow the surface topography even on surfaces with significant roughness and filler-induced heterogeneities. The lamellar, self-assembled morphology observed here for GO-rich regions is consistent with previous automated APPJ depositions of GO [35,38], where micron-scale lamellae coalesce into continuous thin or thick films depending on the deposition time. This conformal coverage across rough, composite substrates aligns with previous APPJ studies on TiO_2_-coated polymeric textiles [36], confirming that atmospheric pressure plasma jets can form adherent oxide layers without requiring high-temperature treatments. Moreover, Del Sole et al. [38] demonstrated that hybrid nanocomposite coatings, combining TiO_2_ and carbon-based nanomaterials, deposited at atmospheric pressure can retain photocatalytic activity while conformally following the underlying microtexture, a behavior qualitatively similar to that of the TiO_2_/GO coatings obtained here. It should be noted that, in the present work, the coating thickness, TiO_2_/GO interlayer structure, and composition gradients were not directly quantified. Therefore, the discussion is restricted to plan-view morphological continuity rather than detailed cross-sectional mapping.

**Figure 13:**
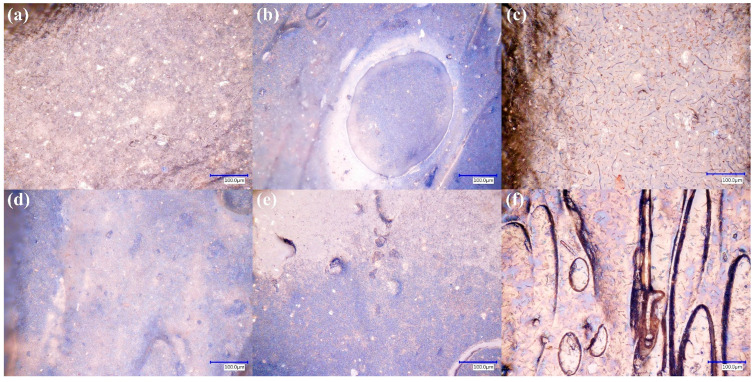
Optical micrographs (500×) of coated PMMA filaments containing carbon black (CB). (**a**–**c**) PMMA with 0.5 wt% CB and (**d**–**f**) PMMA with 1 wt% CB after APPJ deposition of (**a**,**d**) TiO_2_/GO, (**b**,**e**) TiO_2_, and (**c**,**f**) GO.

Similar behavior is observed for the PMMA/CB composite filaments (Figure 13). The first row corresponds to filaments with 0.5 wt% CB, while the second row corresponds to 1 wt% CB. In both cases, the TiO_2_ coatings (Figure 13b,e) produce a bright band that follows the filament curvature, and the transition between the coated and uncoated regions is shown in Figure 13e. The GO layers (Figure 13c,f) again impart an iridescent contrast that overlays the darker PMMA/CB substrate. Despite the different CB loadings, the overall coating continuity appears similar, suggesting that within the studied range, the filler concentration does not strongly hinder film formation.

When Figure 12 and Figure 13 are considered in conjunction with the uncoated morphologies (Figure 6, Figure 7, Figure 9 and Figure 11), it becomes evident that the APPJ coatings conformally cover substrates with very different topographies: grain-like ABS compared with more layered PMMA, and smoother GF-filled contrasted with rougher CB-filled surfaces. This conformal coverage on curved, filler-rich filament geometries is particularly relevant for prospective photocatalytic and sensing applications, where both light exposure and liquid–solid contact occur on complex 3D surfaces rather than on flat coupons.

Although photocatalytic tests are beyond the scope of this work, the TiO_2_/GO coating structure and the wettability changes induced by APPJ treatment align with design guidelines for semiconductor photocatalysts, where band-edge positions, surface states, and interfacial transport jointly control radical generation at the solid–liquid interface [39]. In this context, the present filaments provide a mechanically robust platform for engineering tailored semiconductor stacks according to the conceptual frameworks discussed by Olea et al. [39].

The intimate contact between TiO_2_ and GO in the APPJ-deposited layers is expected to promote charge separation and interfacial electron transport, as reported for graphene-modified TiO_2_ composite photocatalysts [36,38,40]. Lo Porto et al. [41] reported that hybrid TiO_2_-based nanocomposite coatings deposited by aerosol-assisted atmospheric pressure plasmas retained high photocatalytic activity while conformally coating complex substrates, supporting the suitability of APPJ-derived coatings for similar applications. In addition, the possibility of post-deposition thermal or hydrogen-assisted reduction of the GO component has already been demonstrated for APPJ-deposited GO films [42], indicating a route to tune the conductivity of the present coatings without modifying the filament bulk.

### 3.4. Wettability of Composite Surfaces

**Figure 14:**
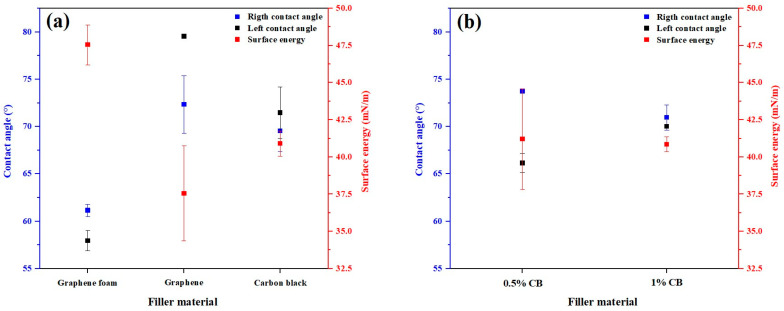
Effect of carbonaceous filler on the wettability of uncoated composite filaments. (**a**) Static contact angle and surface energy of ABS filaments filled with 1 wt% graphene foam (GF), graphene (G), or carbon black (CB). (**b**) Corresponding data for PMMA filaments with 0.5 and 1 wt% CB, showing the narrower contact angle range and minor sensitivity to CB loading.

The wettability of the ABS and PMMA composites was first evaluated in the absence of APPJ coatings (Figure 14). For ABS-based filaments filled with 1 wt% GF, graphene, or CB (Figure 14a), the static contact angles predominantly fall within 60–80°, with noticeable differences among the fillers. Graphene-filled ABS exhibits the highest contact angles and the lowest calculated surface energy, indicating a more hydrophobic character. In contrast, GF-filled and CB-filled ABS composites exhibit slightly lower contact angles and higher surface energies, indicating that the specific filler morphology (3D foam vs. particulate) and its interaction with the ABS matrix influence the liquid–solid interaction. These results show that even before surface modification, the choice of carbonaceous filler can be used to tune the baseline wettability of the filaments. In the following analysis, the static contact angle θ is used as a practical proxy for surface free energy. A decrease in angle is interpreted as an increase in liquid–solid affinity and a higher effective surface energy, whereas an increase indicates reduced wettability and a lower effective surface energy. Considering the moderate roughness levels observed in the optical and SEM micrographs (Figure 4, Figure 5, Figure 6, Figure 7, Figure 8, Figure 9, Figure 10 and Figure 11), the measured contact angles are expected to exhibit predominantly Wenzel-type behavior, in which surface roughness amplifies the intrinsic wetting tendency imposed by the underlying polymer–filler matrix and the APPJ-deposited coating. Such roughness-amplified wetting behavior is consistent with the superhydrophilic states commonly observed on plasma-treated polymer surfaces [43,44].

For PMMA-based composites (Figure 14b), the influence of CB content is more modest. Filaments containing 0.5 and 1 wt% CB exhibit contact angles in a narrower range, and the corresponding surface energies remain similar within experimental uncertainty. A noticeable gap between the left and right contact angles is observed for the 0.5 wt% CB composition, which can be attributed to local surface irregularities and small misalignments during droplet placement. Overall, Figure 14 indicates that, unlike ABS, PMMA’s intrinsic surface dominates the wetting response and CB mainly acts as a pigment and roughness modifier rather than as a strong wettability driver.

**Figure 15:**
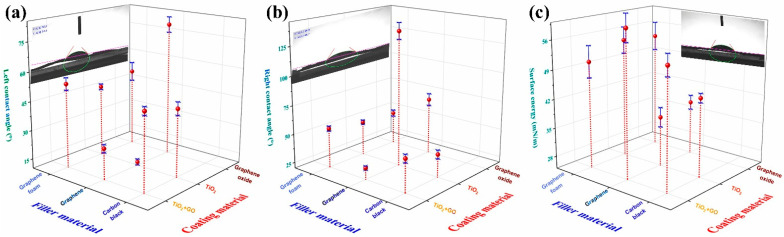
Combined influence of filler and coating on the wettability of ABS composite filaments. Three-axis plot summarizing (**a**,**b**) left/right static contact angles and (**c**) surface energy for ABS filaments filled with GF, G, or CB and coated by APPJ with TiO_2_/GO, TiO_2_, or GO.

The impact of TiO_2_- and GO-based coatings on ABS composites is summarized in Figure 15. The three-axis representation compiles, for each filler and coating combination, the left and right static contact angles and the corresponding surface energy. Compared with the uncoated case (Figure 14a), the coatings markedly broaden the range of contact angles, spanning from relatively hydrophilic values (≈40–50°) to more hydrophobic regimes (above 90°), depending on the filler–coating pair. TiO_2_-only coatings tend to occupy the upper end of the surface-energy scale, which is consistent with their oxide nature and expected affinity for polar liquids. However, local surface roughness and coating thickness variations lead to significant left–right asymmetries, especially in GF-filled composites, where protrusions and grooves inherited from the filament (Figure 9) amplify minor differences in local chemistry into measurable contact-angle differences.

**Figure 16:**
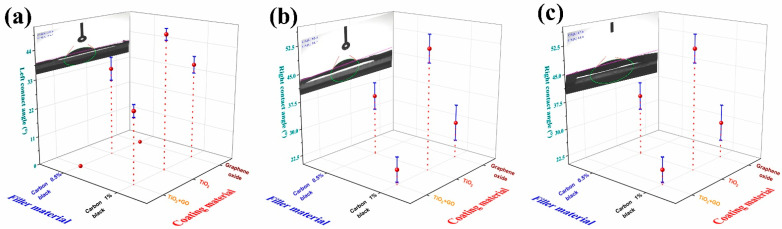
Effect of TiO_2_- and GO-based coatings on the wettability of PMMA/CB composite filaments. Three-axis plots of left/right static contact angles and surface energy for PMMA filaments filled with 0.5 and 1 wt% CB and coated with (**a**) TiO_2_/GO, (**b**) TiO_2_, and (**c**) GO by APPJ.

Figure 16 presents an analogous three-axis analysis for PMMA/CB composites with 0.5 and 1 wt% CB coated with TiO_2_/GO, TiO_2_, or GO. Across all cases, contact angles range from approximately 15° to 125°, while surface energies cluster around ~50 mN/m. These results confirm that the APPJ coatings can substantially alter the wettability of PMMA-based filaments, despite the limited effect of CB alone (Figure 14b). TiO_2_-containing coatings (TiO_2_ and TiO_2_/GO) generally yield higher surface energies and lower contact angles than GO-only coatings, which is consistent with a more hydrophilic behavior. The difference between 0.5 and 1 wt% CB is small compared to the effect of the coating chemistry, indicating that, for PMMA, surface modification dominates over bulk filler concentration in determining wettability.

A direct comparison of Figure 15 and Figure 16 shows that ABS/GF composites with TiO_2_- or TiO_2_/GO coatings achieve some of the highest contact angles and pronounced left–right asymmetries, reflecting the combined influence of a highly structured filler and a conformal, but locally uneven, oxide layer. In contrast, PMMA/CB composites display more symmetric contact angles and a narrower dispersion in surface energy, consistent with their smoother cross-sectional morphology (Figure 7) and less pronounced crust formation. Notably, the combination of filler selection (Figure 4, Figure 5, Figure 6, Figure 7, Figure 8, Figure 9, Figure 10 and Figure 11) and APPJ coatings (Figure 12 and Figure 13) provides a tunable platform for designing surfaces that range from moderately hydrophilic to strongly hydrophobic within the same waste-polymer-based system. This is relevant for applications where controlled liquid spreading, droplet mobility, or coating adhesion are critical, such as photocatalytic reactors, microfluidic devices, or soft robotic components. Recent work by Chen et al. [26] has shown that 3D-printed TiO_2_-based reactors fabricated using DLP can achieve high photocatalytic efficiencies when the geometry and solid loading are optimized, aligning with the envisioned use of the surface-engineered filaments as architected photocatalytic supports.

From a wetting-physics standpoint, the broad distribution of apparent contact angles and the pronounced left–right asymmetries observed, particularly for ABS/GF coated with TiO_2_-based layers (Figure 15), are consistent with roughness-amplified wetting in the Wenzel and Cassie–Baxter regimes. In these regimes, chemically hydrophilic oxide coatings deposited by APPJ can still yield high apparent contact angles when droplets partially rest on air pockets or are strongly pinned at asperities inherited from the composite filaments. This interpretation aligns with the morphological observations in Figure 6, Figure 7, Figure 9 and Figure 11, explaining why filler/coating combinations that maximize multiscale roughness also exhibit the highest contact-angle hysteresis and asymmetry. Such combinations of high surface energy, large hysteresis, and roughness-induced pinning in these APPJ-treated composites are consistent with the plasma-induced formation of hydrated, nano-rough surface layers described by Mozetič [43] for various polymer substrates. They are typical of plasma-engineered surfaces designed for controlled wetting and liquid management [44], which can be exploited in future microfluidic or photocatalytic reactor designs based on these filaments.

Although quantitative pull-off or cross-cut adhesion tests were not performed on the coated filaments in this study, no cracking, peeling, or delamination of TiO_2_, GO, or TiO_2_/GO layers was observed during sample handling, contact-angle measurements, or FDM feeding. These qualitative observations are consistent with previous APPJ works on flat polymer substrates processed under similar plasma conditions, where TiO_2_- and GO-based coatings achieved adhesion levels compliant with ASTM D4541 and D3359 protocols [32]. Future work can extend this analysis to filament geometries to quantify coating–substrate adhesion as a function of filler distribution, roughness, and coating configuration.

### 3.5. Calorimetry and Thermal Stability

**Figure 17:**
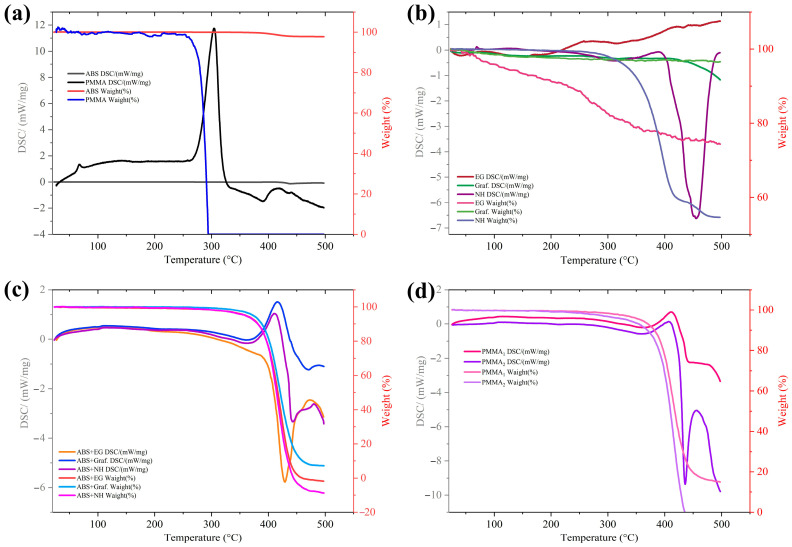
Thermal behavior of polymers, fillers, and composites obtained from simultaneous TGA/DSC analysis. (**a**) Pure ABS and PMMA, showing distinct degradation and glass-transition/melting events. (**b**) Carbonaceous fillers (graphene, graphene foam, and carbon black), evidencing the high thermal stability of graphene relative to CB and GF. (**c**) ABS composites with GF, G, and CB, whose degradation onsets are similar to those of neat ABS but with modified crystallization and vitrification peaks. (**d**) PMMA composites with 0.5 and 1 wt% CB, which display earlier mass loss and altered transition temperatures compared with PMMA. Dotted lines indicate the horizontal as an eye guide.

Figure 17 summarizes the thermal behavior of the polymers, fillers, and composites obtained from simultaneous TGA/DSC analysis. The pure polymers (ABS and PMMA) are shown in Figure 17a. PMMA exhibits a two-step mass loss, starting around 280 °C and stabilizing near 345 °C, corresponding to depolymerization and fragmentation. ABS begins to lose mass at slightly lower temperatures (≈270 °C) and exhibits a more continuous mass loss, reaching nearly complete degradation by the end of the test. The associated DSC curves reveal a first melting or softening event for ABS below 200 °C, followed by a glass transition near 231 °C and a crystallization/reorganization peak around 371 °C, before decomposition accelerates above 400 °C. PMMA, in contrast, presents two main endothermic events at approximately 305 °C and 374 °C, consistent with the onset and progression of its thermal degradation.

The thermal responses of the isolated carbonaceous fillers are shown in Figure 17b. Graphene exhibits the highest stability, with a total mass loss of only about 3% over the explored temperature range, in agreement with previous reports on graphene-based systems [45]. Carbon black begins to lose mass at approximately 236 °C, with a pronounced step near 421 °C, and a final mass loss of roughly 65%. GF exhibits an initial 2% mass loss at ~141 °C, which increases gradually to ~10% at higher temperatures. This can be attributed to the release of trapped solvents, functional groups, or residual foaming agents. A modest exothermic event associated with carbon black crystallization is observed near 446 °C. Overall, these results confirm that the fillers are thermally stable under typical FDM processing temperatures (<250 °C) and that graphene, in particular, can act as a stabilizing component in the composite.

Figure 17c presents the TGA/DSC curves for ABS composites containing GF, graphene, or CB. All three formulations exhibit a similar onset of mass loss at around 264 °C and almost complete mass loss at high temperatures, indicating that the presence of carbonaceous fillers does not significantly alter the ultimate thermal degradation of ABS. However, subtle differences in the DSC peaks are evident. ABS/graphene and ABS/CB exhibit melting-like events near 410–415 °C and vitrification peaks at around 470 °C and 443 °C, respectively, similar to those of neat ABS. In contrast, ABS/GF exhibits additional features: a glass transition at approximately 265 °C, another transition near 321 °C, and a crystallization peak around 428 °C. This altered thermal signature suggests that the 3D structure of GF perturbs the packing and relaxation dynamics of the ABS chains more strongly than particulate fillers do, which may contribute to the distinct morphology and crust formation observed in Figure 11.

The PMMA composites with 0.5 and 1 wt% CB are shown in Figure 17d. Both formulations exhibit an earlier onset of mass loss (≈234 °C) and nearly complete material loss at the end of the test, in contrast to the more gradual degradation of pure PMMA. The DSC curves reveal melting peaks at 412 °C and 406 °C for 0.5% and 1% CB, respectively, followed by glass-transition-like events at 457 °C and 437 °C. The slightly higher energy release observed for the 1 wt% CB filament is consistent with a higher carbonaceous content and qualitatively agrees with previous studies on ternary PMMA composites containing carbon fibers and CB [5]. In line with the ABS results, GF-containing ABS filaments exhibit higher energy release than GF alone or pure ABS [46], indicating an interplay between the foam-like filler and the host matrix.

Figure 17 confirms that the waste-derived ABS and PMMA composites with carbonaceous fillers can be safely processed by extrusion and APPJ at the temperatures used in this work. The fillers do not significantly compromise the thermal stability of the polymers, and in some cases (graphene, GF), they introduce additional relaxation and crystallization features that may be exploited to tune dimensional stability and heat management in 3D-printed parts. This thermal robustness underpins the morphological (Figure 4, Figure 5, Figure 6, Figure 7, Figure 8, Figure 9, Figure 10 and Figure 11) and wettability (Figure 14, Figure 15 and Figure 16) results, supporting the use of these composites as functional filaments for additive manufacturing. Similar processing windows and thermal stability were reported for ABS–graphene filaments designed for FDM [12], supporting the idea that low-to-moderate carbon loadings can be used to tune functional properties without severely narrowing the usable temperature range.

From a processing standpoint, these results indicate that the selected filler loadings do not narrow the thermal range available for filament extrusion and APPJ treatment, while the high stability of graphene and the subtle GF-induced changes in ABS relaxation and crystallization behavior can be exploited to manage heat dissipation and dimensional stability in printed parts.

## 4. Conclusions

This work explored a sustainable route to produce multifunctional filaments by combining waste polymers, carbonaceous fillers, and APPJ-deposited oxide/graphene-oxide coatings. The main conclusions are:

1. ***Waste thermoplastics as viable matrices for composite filaments***

Ground ABS waste and commercial PMMA can be compounded with carbon black, graphene, or graphene foam and extruded into continuous filaments. CB, G, and GF disperse reasonably uniformly at the microscale, acting as pigments and structural modifiers without preventing filament formation.

2. ***Filler-dependent morphology in implications***

Digital microscopy and SEM showed that PMMA/CB filaments transition from smooth particles to pigmented strands, exhibiting increased surface roughness and reduced large-scale porosity as the CB loading increases. ABS composites, in contrast, reproducibly develop a carbon-rich outer crust surrounding a lighter ABS core in the cross-sections examined under the present extrusion conditions, with GF generating the darkest and most heterogeneous pre-extrusion mixtures. These matrix- and filler-dependent morphologies are expected to impact mechanical response, heat dissipation, and interfacial behavior.

3. ***Conformal APPJ coatings on complex composite substrates***

APPJ deposition of TiO_2_, GO, and TiO_2_/GO produced continuous coatings that conformally follow the curved and rough filament geometries of ABS and PMMA composites, despite pronounced differences in roughness, filler content, and cross-sectional texture. Uncoated ABS composites already exhibit filler-dependent wettability, whereas PMMA/CB filaments display a narrower contact-angle window. After APPJ treatment, TiO_2_- and GO-based layers broaden the accessible contact angle range (≈15–125°) while maintaining surface energies around 50 mN m^−1^, providing a means to tune wetting behavior without altering the underlying bulk formulation.

4. ***Thermal stability and printability range***

TGA/DSC analyses confirmed that ABS and PMMA composites retain thermal stability within the processing range used for extrusion and APPJ treatment. Graphene shows the highest intrinsic thermal stability among the fillers, while GF subtly modifies the relaxation and crystallization behavior of ABS. FDM trials demonstrate that ABS-based composites with CB, G, or GF can be reliably fed and printed into stepped 3D prototypes with good agreement between CAD and final geometries. By contrast, PMMA composites are too brittle for continuous FDM under the tested conditions, underscoring the need to adjust formulation and processing parameters if PMMA-based waste streams are to be used as structural feedstocks.

5. ***Outlook for circular, surface-engineered polymer manufacturing***

The combination of waste-polymer reuse, tailored carbonaceous fillers, filament-scale morphological control, and APPJ TiO_2_/GO coatings defines an integrated platform for surface-engineered filaments. Within this platform, recycled ABS/PMMA cores provide mechanical strength and thermal robustness, while plasma-derived coatings locally tune wettability, interfacial adhesion, and potential photocatalytic response. This modularity is particularly attractive for circular manufacturing routes targeting 3D-printed microreactors, microfluidic manifolds, or soft robotic elements, where geometry, filament architecture, and surface chemistry can be co-designed for application-specific performance. Within this modular architecture, the same waste-derived composite core can be paired with different plasma-deposited coatings to independently tune wettability and interfacial chemistry, which is not achievable in bulk-doped TiO_2_–polymer filaments.

## Data Availability

Data is contained within the article. Further inquiries can be directed to the corresponding authors.
